# An Empiric Experience Implementing a Methodology to Improve the Entrepreneurial Support System: Creating Social Value Through Collaboration and Co-creation

**DOI:** 10.3389/fpsyg.2021.728387

**Published:** 2021-10-22

**Authors:** Jokin Cearra, María Saiz-Santos, Jon Barrutia

**Affiliations:** ^1^Cámarabilbao University Business School - Licenciado Poza, Bilbao, Spain; ^2^Financial Economy II, University of the Basque Country (UPV/EHU) - Lehendakari Aguirre, Bilbao, Spain

**Keywords:** entrepreneurship, entrepreneurial ecosystem, entrepreneurial support system, network, co-creation, social value, stakeholders, information and communication technologies (ICTs)

## Abstract

Entrepreneurs are considered an important source of innovation, acting as agents of change in developed societies. For entrepreneurs to develop, entrepreneurial ecosystems are required. These environments are complex heterogeneous systems. However, the atomization of the subsystem of agencies and organisms supporting entrepreneurial activity can cause problems. To solve this governance problem, a social experiment was designed to test the value of a solution based on a technological platform. The methodology is based on a dynamic scheme, seeking the involvement and collaboration of all the stakeholders. This method uses a co-creative process inspired by design thinking. The theoretical framework included literature on entrepreneurial ecosystems and governance theory and took into account the need to involve all the stakeholders to improve the previous situation and achieve sustainable development goals. Based on the application and an *ad-hoc* methodology seeking the involvement and collaboration of all stakeholders, a social network supported by an ICT-based platform was formally created, contributing to alleviate the atomization problem and generating social value at the same time. This social experiment, carried out in the Spanish province of Biscay in the Basque Country, was a pilot test and could be extended to other entrepreneurial ecosystems with similar casuistic frameworks.

## Introduction

Creating new companies allows an economy to grow and generates employment. It constitutes one of the main sources of innovation and is, therefore, one of the key elements of competitiveness in a globalized market. However, entrepreneurship is not just an economic phenomenon, as outlined by Korsgaard and Anderson (2011), who explore beyond purely economic results, analyzing the social outcomes created by the entrepreneurial process. They argue that social factors play a role at different levels, and act as an enabler and the context of the entrepreneurial activity. They create different types of social values, depending on both the type of project and the level analyzed, from individual to societal contexts (Korsgaard and Anderson, 2011), all of which are interrelated in complex ways.

Innovation is one of the outcomes of entrepreneurial endeavor, and also a way of searching for solutions to the challenges and problems humanity is facing. They are synthesized in the 2030 Agenda with 17 sustainable development goals (SDGs) related to innovation. The Global Entrepreneurship Monitor (GEM) (Kelley et al., 2012) highlights the importance of the environment in fostering entrepreneurial activity and, thus, contributes to economic development. To achieve this, it is not enough to create a favorable institutional framework, there also needs to be an interrelation and collaboration between entrepreneurs, organizations, and the different agents of the environment. These relationships shape what Mason and Brown (2014) call the entrepreneurial ecosystem. This new concept offers a systemic view of entrepreneurship (Cavallo et al., 2019), but not as a simple system; rather, entrepreneurship is complex and dynamic (Isenberg, 2010; Feld, 2012; Spigel, 2017; Han et al., 2019). The whole ecosystem is formed by different interconnected subsystems (Motoyama and Knowlton, 2017) and due to the aforementioned complexity, Miller and Acs (2017) suggest going through its parts instead of pretending to reach an integral modelization. One of these subsystems is the support system, which is composed of various members who share the same goal of entrepreneurial support within a local geographic community (Theodoraki et al., 2018). In many ecosystems, there is a conglomerate of public and private agents covering the different areas that an entrepreneurial project goes through. Motoyama and Knowlton (2017) apply the social network perspective to analyze the relationships and interactions between and among entrepreneurs, support organizations, and other secondary support actors, to uncover what is happening in the case of St. Louis. They are of the view that previous research has considered this subsystem as a black box since studies tend not to clarify the connections and interactions inside the subsystem and ecosystem. One of their findings is that the ways in which support organizations interact significantly impact how and why entrepreneurs connect (Motoyama and Knowlton, 2017).

Hayter (2016) considers that the interactions in the different networks conform to an ecosystem that evolves in time, as it is a dynamic entity. The entrepreneurs arrive at the resources through the relationships and interactions of their networks, so their social capital is a critical element. Colombelli et al. (2019), describing the entrepreneurial ecosystem of Turin (Italy), say that the system is characterized by its high entropy. The support subsystem is formed by a wide variety of actors, but there is a lack of teamwork, overall, among public entities.

This need for better coordination has been addressed in different countries. In the United States, the U.S. SourceLink program (Meyers, 2011) is incorporated for this cause. It connects entrepreneurship support organizations to the entrepreneurs whom they serve, to leverage their resources and improve their services. This initiative was born in Kansas City in the late 1990s, as it was perceived that many organizations would provide entrepreneurial support services, but aspiring and existing business owners were unable to find the right examples. The first step was to create a one-stop-shop for entrepreneurs, which joined several organizations in the same physical location. However, the idea soon evolved into creating a network to link partners. Consequently, any entrepreneur connecting to the network could refer to the right resources for their needs and stage of business instead of being shuffled from place to place. This network is supported by an internet-driven simple point and a click engine called The Resource Navigator. There are networks in more than 20 regions in the country.

The same obstacle to growth and entrepreneurship in Spain lies in so-called atomization. The existence of a large number of simultaneous programs and organizations, as well as the overlapping discoordination of their actions, means potential entrepreneurs are often not clear where to go. According to the GEM's special report about organisms and measures, this lack of support for entrepreneurs in Spain is an extended problem (Rubio and Sánchez, 2016).

In the specific case of the Basque Country, the GEM 2018 Report (Saiz et al., 2018) concluded that public policies are the main positive driver in supporting and promoting entrepreneurial activity. Despite recognizing its effectiveness, the report identifies the complexity of bureaucratic processes, low efficiency of unique windows, and demand for greater institutional coordination, as obstacles to entrepreneurship.

One of the problems our society faces is the complicated entry of young people into the labor market. The complexity of this transition means that their talent, along with the capital and resources invested in their preparation are not being taken advantage of, as these collective concerns and business ideas could become viable entrepreneurial projects. It should also be taken into account that many young people have a sustainable approach to projects in addition to a purely entrepreneurial one. Apart from these economic results, they take into account social and environmental factors, known as the triple bottom line (Elkington, 1994). Therefore, supporting young people enables increased social cohesion. It is up to institutions to help and facilitate young people in the development of business projects and the startup of their companies, which they do. Numerous agents and agencies support new ventures in the Basque province of Biscay. With the challenge of improving the functioning and coordination of the agents of the subsystem of support, a project based on the collaboration of all the components of the ecosystem of the province was carried out during the first term of 2018.

The initial objectives discussed in this research project at its initiation were:

a) To detect the needs of young entrepreneurs.
b) To obtain the involvement of the agents of support to improve the coordination, collaboration, and operation of the proper network.
c) The creation or definition of a shared entity such as a portal or online platform that gathers and connects the young entrepreneurs of Biscay with support agents, providing services with added value and improving the interconnection between them.

To achieve these objectives, we developed a methodology (Balderas et al., 2020) based on co-creation and design thinking (Brown, 2008), with the participation of the full range of stakeholders. The process was divided into three phases:

1) The first sought collective reflection and the promotion of the spirit of collaboration between people and institutions related to entrepreneurship, to build a network based on collaboration.
2) The second had the objective to build together and generate ideas to center them through a process of co-creation.
3) The third and last session objective was to land the proposals generated in the second one.

This kind of effort has importance and creates social value from several points of view. First, as Agrawal et al. (2015) outline, the value emerging in co-creation is social and should be appreciated in a social context, where a large number of stakeholders are present in the system. Second, new governance models are needed to align the functioning and objectives of public and private stakeholders. Third, because of attempts to improve the entrepreneurial ecosystem to favor entrepreneurial activity, producing new companies and employment, which are essential for innovative projects to flourish; and fourth, because innovation is crucial for accomplishing SDGs in the long term.

This paper presents an experience of collaboration between agents of the entrepreneurial ecosystem in Biscay, who worked together to improve the entrepreneurial project's support subsystem. As the solution was not planned at the beginning and arose during the planning process, a longitudinal study analyzing the development and effect on the whole system might be required. Alvedalen and Boschma (2017) performed a literature review and found many static approaches that merely described the relations in the entrepreneurial ecosystem, indicating that there is a poor understanding of how it evolves and by which process it develops over time. Therefore, this experience might be used as a pilot test—since atomization and a lack of coordination are problems affecting the entrepreneurial environment in several ecosystems—but also as a longitudinal embedded case study of the evolutionary dynamics of a specific entrepreneurial ecosystem.

## Theoretical Background

### Productive Entrepreneurship, Social Value, and Stakeholders

Entrepreneurs have a high potential for generating social, productive, and cultural changes in the regions where they act. Literature on entrepreneurship confirms that they are agents of change, innovation, employment, and the creation of new businesses. Consequently, entrepreneurs are a necessary part of our societies, especially when the community is facing new economic and social scenarios. Entrepreneurship plays an increasingly important role in achieving economic growth and progressing innovation (Acs, 2006; Audretsch et al., 2006; Minniti, 2008; Wennekers et al., 2010; Stam, 2015). However, as Baumol (1996) expounds, the exercise of entrepreneurship is not always productive; it can at times be unproductive or even destructive, depending on the structure of payoffs in the economy that constitute the rules of the game. In addition, focusing specifically on social value creation, Acs et al. (2013) define productive entrepreneurship as creating both social and economic value, while unproductive and destructive entrepreneurship generates economic value for the entrepreneur but does not result in net social value creation.

Mazzucato (2018) also focuses on the difference between productive and unproductive entrepreneurship. Moreover, apart from questioning the role of the different actors in the innovative movement, Mazzucato makes a distinction between value creation (and its destruction) and value extraction, questioning the measurement of that value. If a value is based on profit as the bottom line of an income statement, several factors are missing, contributing to undesirable societal situations. As outlined in the preface of the book “if the goal is to produce growth that is more innovation-led (smart growth), more inclusive and more sustainable, we need a better understanding of value to steer us” (Mazzucato, 2018, p. 11). Furthermore, this study explains how the paradigm passes from maximizing shareholder value to take into account stakeholder value.

Mazzucato refers to the value in terms of the process by which wealth is created, considering it a flow (2018). This flow results in actual things, whether tangible (a loaf of bread, for example) or intangible (new knowledge), and wealth, instead, is regarded as the cumulative stock of the value already created. In this way, this study defines value creation as to how different types of resources (human, physical, and intangible) are established and interact to produce new goods and services. Mazzucato considers value extraction activities focused on moving around existing resources and outputs, which gain disproportionately from ensuing trade (2018).

Additionally, Kuratko et al. (2017) analyze the social value creation in business, defined as creating benefits beyond those captured by their creator. They explain that in recent years, there has been a decided increase in the emphasis on social value creation by all organizations, including for-profit organizations for several reasons: (1) customers want to buy from these companies, (2) employees want to work for them, (3) investors are willing to invest in them, and (4) entrepreneurs hope to start them (Kuratko et al., 2017). However, despite rapidly growing interest in social entrepreneurship, there is no equivalent instrument regarding social value creation.

Even though corporate social responsibility (CSR) has been studied for quite some time, CSR scholars tend to focus more on protecting the business's social license to operate in society by developing goodwill, rather than identifying opportunities to create, deliver, and capture social value (Bansal and Roth, 2000; Auld et al., 2008). More significantly, if businesses are going to emphasize social value more, managers and employees will likely need to monitor the environment and continually revisit the ways they create, deliver, and capture social value, just as they have done with financial value. New instruments are needed to measure individual perceptions of the current organizational environment to determine whether it is conducive to individual efforts to create social value.

Stakeholder theory was developed by Freeman (1994). It states that stakeholders are defined as any group of individuals who can affect, or be affected, by the achievement of the organization's objectives and considers that the integration of the interests of all stakeholders is necessary to generate sustainable profits in the long term. Despite Freeman's (1994) broad definition, stakeholders could be groups of individuals that are important for the success of the organization. Curiously, stakeholders could be the same using either Freeman (1994) or Friedman's (1962) theory, the latter of which states that the objective should be to maximize the shareholder value: owners, workers, suppliers, clients, and the local community.

As Retolaza et al. (2009) suggest, the link between stakeholder theory and CSR is close and based on those concepts on which they develop a model to apply newly created firms. Their proposal focuses on the inclusion of social responsibility as a new factor, under the value innovation model proposed by Kim and Mauborgne (2005). An important aspect to introducing this new factor relies on the necessity to align it with the strategy, which will use the balanced scorecard model developed by Kaplan and David (1992).

Focusing on society, entrepreneurial abilities and attitudes, risk-taking behavior, and creativity are crucial competencies in the development of economies (Guellec and Wunsch-Vincent, 2009). That is why scholars have an increasing interest in measuring entrepreneurship and studying the role of specific environments in different countries to promote these competencies and new business creation (Mason and Brown, 2014).

### Entrepreneurial Ecosystem

As stated, entrepreneurship is not an isolated phenomenon. It occurs due to—and is affected by—the conditions present in the environment. Entrepreneurial activities are the result of the decision of the players according to the institutional framework that sets the rules of the game. North (1990) divides those rules (or institutions in terminology) into formal (regulations, laws, policies, and agencies) and informal (culture, beliefs, values, ideas, habits, and attitudes of society) factors.

Entrepreneurs are not isolated players taking their decisions and pursuing their ventures, meaning their activity can be seen as a system or network of interconnecting and interacting parts, which is the definition of an ecosystem. Mason and Brown (2014) define an entrepreneurial ecosystem as:

“a set of interconnected entrepreneurial actors (both potential and existing), entrepreneurial organizations (e.g., firms, venture capitalists, business angels, banks), institutions (universities, public sector agencies, financial bodies), and entrepreneurial processes (e.g., the business birth rate, numbers of high growth firms, levels of ‘blockbuster entrepreneurship’, number of serial entrepreneurs, degree of sell out mentality within firms and levels of entrepreneurial ambition) which formally and informally coalesce to connect, mediate and govern the performance within the local entrepreneurial environment” (Mason and Brown, 2014, p. 82).

Based on their previous research, Stam and Spigel (2016) undertook a critical review of the literature in this field to reach a definition of the term and an integrative model. They define an entrepreneurial ecosystem as a set of interdependent actors and factors coordinated in such a way that they enable productive entrepreneurship in a particular territory (Stam and Spigel, 2016). Productive entrepreneurship is any entrepreneurial activity that contributes directly or indirectly to new outputs in the economy or the capacity to produce new outputs (Baumol, 1993). In their approach, the government is not the leader but a feeder of the ecosystem that creates the proper economic, social, and legal environment that enables new ventures to flourish (Stam and Spigel, 2016). Additionally, startups are at the center of the ecosystem, but entrepreneurs might also act as feeders or facilitators, as both mentors and models for new players.

Mazzucato (2011) expands the scope of these ideas, outlining that the state plays roles in entrepreneurial activity in broader ways than are formally recognized. For example, by providing public funds at the beginning, they support the most uncertain phase of research, when the private sector might not be willing to invest due to the risks involved. This should be taken into account when debating the role of government in the future. Additionally, in 2021, Mazzucato developed the idea of the need to use new forms of collaboration between public and private actors to build more mutualistic and symbiotic collaboration systems. As stated in the previous section, it is necessary to use stakeholders' points of view as well as shareholders, but all actors must be rewarded: workers, communities, and the environment.

Based on best practice and research, Isenberg (2010) sets out nine principles for building an entrepreneurial ecosystem that government leaders should focus on. They suggest that instead of trying to replicate Silicon Valley, a region should shape the ecosystem around local conditions (Isenberg, 2010), and the private sector should be engaged from the start, since the government cannot build the ecosystem alone. Additionally, it is better to favor high growth, since its impact and influence as a reference and inspiration is better than encouraging self-employment. This takes us to the next point, which is to use the big successful experiences as an example and bring them to the public, even over-celebrating success (media events, highly publicized awards, speeches, etc.). Cultural change is also important in changing minds, and apart from the precedent factor, the media plays an important role. As opposed to focusing on easy money, it is preferable to “stress the roots” and bring out the imagination and innovation from entrepreneurs, as even startup incubators have been proven to be not good enough to develop strong companies. Facilitating an entrepreneurial environment is not a matter of generating clusters of innovation artificially; it is better if they generate and grow organically with support from the government. Isenberg (2010) finishes by saying that legal, bureaucratic, and regulatory frameworks should be revised. The latter is what most governments focus on but a holistic view with all the precedent principles is required.

However, it is also true that to create an ecosystem, encouraging entrepreneurs and learning from failures might encourage new initiatives to flourish in different countries. There are many different approaches or policies to enhance entrepreneurial activity such as education, and labor market regulation, etc., instead of thinking solely about entrepreneurial public policy.

Public policies that are only focused on economic promotion are not enough to consolidate a successful entrepreneurial ecosystem. They contribute in many ecosystems to the above-explained atomization of agents, providing services and assistance to entrepreneurs in an uncoordinated way. That questions the efficiency of public efforts invested. According to Spigel (2017), the support system is one of many core components of an entrepreneurial ecosystem, but as Motoyama and Knowlton (2017) remark, it is considered a black box by most previous research, which has not analyzed how it works.

Mack and Mayer (2016) showed that it is interesting to analyze the interdependence of the elements of the ecosystem. They explore its evolutionary dynamics by taking into account the institutional framework and the sociopolitical context in which it has evolved over time, along with the role of regional policy in acquiring missing elements and facilitating interaction between these and other elements. Mack and Mayer (2016) distinguish four stages of ecosystem development starting from birth, followed by growth, sustainment, and ending with decline. Each of the stages was characterized by a different mix of Isenberg's (2011) entrepreneurial ecosystem domains. Furthermore, Hayter (2016) outlines the evolutionary dynamics of the ecosystem with social networks as critical pathways, through which entrepreneurs access resources and other contacts important for the development of their ventures, social capital being a critical element for the exchange of information and resources, and explaining the evolution through network bridging.

Audretsch and Belitski (2017) define systems of entrepreneurship (as a synonym to ecosystem) as institutional and organizational, along with other systemic factors, such as the educational system. Education is an important tool for stimulating entrepreneurship (Harris and Gibson, 2008; Raposo and Do Paço, 2011). Entrepreneurship research provides evidence that there is a positive and robust link between entrepreneurial education and entrepreneurship performance (The Small Business Economy, 2007).

This section has analyzed entrepreneurial ecosystems and their problems, and the next section will focus on society in a broader sense.

### Sustainable Development Goals and Governance

Sustainable Development Goals (SDGs) set the framework for national action and international cooperation on sustainable development. The United Nations has set 17 goals that to be achieved in the next decade. These ambitious goals summarize the biggest challenges we face, and although they are all interrelated and must be taken into account, within the scope of this project, we highlight the following: promoting constant, inclusive, and sustainable economic growth, and full employment that is productive and decent work for all (decent work and economic growth, Goal 8); to build resilient infrastructure, promote inclusive and sustainable industrialization, and foster innovation (industry, innovation, and infrastructure, Goal 9); promote peaceful and inclusive societies for sustainable development, facilitate access to universal justice and create efficient, inclusive, and accountable institutions at all levels (peace, justice, and strong institutions, Goal 16); and, to strengthen the means to implement and revitalize the Global Partnership for Sustainable Development (partnerships to achieve the goals, Goal 17).

The International Institute for Applied Systems Analysis The World in 2050 Initiative (2018) carried out The World in 2050 report to provide the scientific foundations for the 2030 Agenda. It is based on the assumption that ambitious but ambiguous SDGs are necessary but not sufficient to lead humanity toward long-term sustainable development. The time horizon and scope go far beyond 2030 and will have to be revisited to adjust the SDGs with regard to longer-term socioeconomic and environmental sustainability. Furthermore, it states says that effective and inclusive governance is a central element of sustainability and transformation The World in 2050 Initiative (2018). Such a transformation toward sustainable development will require profound normative and social, political, and institutional changes Sachs et al., 2019. Key elements include investments in capable public institutions, active civil societies, sustainability-oriented partnerships, science, engineering, the private sector and governments, and the formulation of plans and roadmaps to achieve the SDGs and long-term sustainability goals.

Mazzucato (2018) suggests the need for a redefinition of policymaking, in the sense that policy can be addressed to shape a different future: co-creating markets and value. A better economy might be created since markets are outcomes of decisions that are made in business, in public organizations, and civil society. This makes it possible to choose a different path among the many that are available. To do so, a purpose-driven sense has to affect the relationships among the different agents. Mazzucato (2021) characterizes it as a mission economy, which requires a radical change in the core of the business models and value chains. The selection of the mission must stimulate the search for multiple solutions, and according to the author, even if it is directed to a specific objective, this should be broad enough to cover numerous projects that, together, carry out the mission (Mazzucato, 2021). This shared mission is the SDGs and the Change (with capital letter due to the size and importance of the challenge), which require governments to reformulate the structure of relationships among economic agents and society. Additionally, rethinking corporate governance has to be prioritized in the agenda; as previously a company's objective had to pass from shareholder to stakeholder value.

Stakeholder value gives meaning to the interaction between the different economic agents and the creation of value in favor of the common good since the value created is reinvested in a larger group of actors, which includes the community. For the governance of such a system, it is essential to focus on relationships that are established, for example, between public and private actors. Many measures can be introduced, for example, conditionality when granting aid or contracts. Nevertheless, the associations also need to be organized.

The application of a mission-oriented thought requires not only the capacity to adapt but also institutional innovations that create new markets and reconfigure existing ones. It also additionally requires citizen participation. In some countries, involving and engaging citizens in mission design has become a fundamental principle of innovation in the public sector, just as it is in the private sector. There are many positive examples in the consultations and idea-generation processes that led to the establishment of SDGs. The joint design guarantees the social appropriation of the objectives of the missions, ensuring that they exceed the term of office of the politicians in charge.

Participation requires rethinking the future together. That is why it is important to bring together different voices not only to react to a mission but to design it. This requires systems to be open to change and adaptation based on the response received.

Co-creation is a paradigm of relationship based on the creation and joint evolution of value among people with shared interests in collaborative and commitment platforms that arise from ecosystems with diverse capacities and that materialize in concrete experiences, which expand the wealth and well-being of society (Echegaray et al., 2017).

The design thinking methodology (Brown, 2008) promotes the participation of all the stakeholders of an ecosystem to design the key lines of a new model of the relationship among them. It is used to develop people-centered innovation processes, offering a lens through which to observe challenges, detect needs, and propose solutions.

Policymakers in Basque Country have been working for years to build a successful entrepreneurship ecosystem. As a result, we see a multitude of entrepreneurial programs (public and private) promoting entrepreneurship. It is important to note that in Basque Country, policymakers used to define measures of economic promotion to increase the entrepreneurial activity rate, but they can also influence it through many other factors. The GEM Basque Country 2019–2020 report (Saiz et al., 2020) shows that “public policies and public programs” promoting entrepreneurship had an excellent score in the entrepreneurship ecosystem, with “education”—primary and secondary—the worst factor assessed.

In this sense, as Mazzucato (2021) says, the attitude of a mission is to identify problems that can channel collaboration between many different sectors; therefore, it is about structuring policies that can attract many types of organizations that carry out different solutions or projects. To do so, the purpose must be part of the core of corporate governance (also in public entities) and a very broad stakeholder-based perspective for the entire economy must be adopted.

With these discussions in mind, this study aimed to undertake a pilot test, a co-creative experience among all the stakeholders involved to deal with a detected problem.

### Hypothesis

The atomization of the network of agents and entities that support entrepreneurial activity provokes the existence of inefficacy and inefficiency in the use of public resources invested. This is a general problem in the Spanish regional ecosystems (Rubio and Sánchez, 2016). Based on the research about the Spanish situation (ENISA y GEM España, 2018) and the previously detailed American US SourceLink experiences, we assume this is a global problem, and used the case of this regional ecosystem as a pilot approach to develop a new methodology. This objective requires the collaborative effort of all the stakeholders related to the support system, who, through a dynamic process based on design thinking, will look for a solution.

Our hypothesis outlines that this experience will lead to an improvement in the entrepreneurial ecosystem but also introduces a new governance model that joins the efforts of the public and private agents and agencies. It is also addressed to undertake the SDGs both directly, by improving the service to entrepreneurial activity, which is one of the sources of innovation, and indirectly, because it could be an inspirational test of how to engage stakeholders in the search for the solutions to the different problems we have to confront in the future.

As explained above, social value is created under various assumptions: from the societal point of view, by favoring the creation of new companies and employment; from the policy point of view, by involving all the stakeholders in the design of public policies, aligning the interest of public and private entities; and from the ecosystem point of view, creating a favorable framework in which innovation can flourish and which, being adequately oriented, could lead to the achievement of the SDGs in the long term.

## Methodology

As mentioned above, the initial objectives were, first, to detect the needs of young entrepreneurs; second, to obtain the involvement of the agents of support to improve the coordination, the collaboration, and the operation of the proper network; and third, the creation or definition of a shared entity such as a portal or on-line platform that gathers and connects the young entrepreneurs of Biscay with support agents, providing services with added value and improving the interconnection between them.

A key requirement of achieving the ultimate goal of the challenge was to encourage the participation and involvement of all the agents in the entrepreneurial ecosystem. This study aimed to build this through participation, involvement, and commitment, meaning we adopted an inclusive methodology based primarily on design thinking and co-creation. A further explanation of the methodology and the process is described by Balderas et al. (2020). In this process, key points are the development of empathy, the generation of as many ideas as possible, the construction of prototypes, and learning from the implementation of that prototype.

A preliminary phase of initial reflection was raised followed by three working sessions, detailed below. A varied and representative sample not only of agents but all related stakeholders were summoned to sessions, with the objective to gather a plurality of approaches and viewpoints to enrich the work. The capacity of our methodology was limited to no more than 60 participants. In addition, between the different phases, the opinion and contributions of the remaining agents of the ecosystem were obtained by online means, as we have shared the results of each session. Online participants who were physically not present were termed as the contrast group.

The first session took place in January 2018, was titled “Collaborating.” It sought the collective reflection and the promotion of the spirit of collaboration between people and institutions related to entrepreneurship, in order to build a network. Assistants ran the six working groups, and each group was tasked with the objective of seeking maximum representation of the ecosystem without losing sight of equitable distribution with respect to gender. Six questions relating to the current situation were distributed randomly among the six working tables, with the generation of ideas being the main activity.

The second session was held in February with the title “Co-creating,” and its objective was to build together and generate ideas to center them through a process of co-creation. To achieve this we used the World-café format, working on two questions that sought to detect the main reasons for slow progress in each of the seven phases of an entrepreneurial project and generate ideas or proposals to overcome these obstacles. Once again, a wide representation of people, agencies, and institutions that make up the entrepreneurship ecosystem were invited.

The third and last face-to-face session of the project had an objective to land the proposals generated in the second session. This final session took place in March, and it was titled “Landing.” The aim was to shape the concepts that had emerged in the previous working days and facilitate the elaboration of an action plan for the implementation of these ideas in the near future.

## Results

This section summarizes the findings obtained during the different steps.

### Collaborating Stage

The objective of the first session was to generate a climate of collaboration between the different agents. The participants were distributed in six tables; each one was asked to complete a representation of the stakeholders of the ecosystem. The issues were worked through according to a prefixed scheme, which tried to diagnose the situation of the question in the province of Biscay (whether in the Basque Country or in Spain or in the North of Spain). The main topics that arose about each theme were:

1) Current situation of the entrepreneurial ecosystem

Here, a lack of clarity was emphasized, both for the entrepreneur and the proper agents/entities of support to entrepreneurship, with regard to who was available and what did they offer, referring to their programs and activities. Thus, the problem labeled as atomization was diagnosed and identified by the participants from the very beginning.

Other ideas were suggestions, but they led to underlying problems. They were related to encouraging an investment culture that favors the financing of entrepreneurship since it was considered another important dearth; a better understanding of the causality of the venture, to better conform to the needs of each case; and the need to adapt the measurement of the results of the agencies to not only take into account the number of companies created under their umbrella but also to open the evaluation to other indicators. This follows with Mazzucato's (2021) suggestion to align the control measures with the objectives of the mission to be accomplished.

2) Needs of the young entrepreneur

The problems derived from atomization were also asserted since there were comments regarding doubts relating to citizenship and about where to apply to receive different kinds of help/support from funding institutions, support agencies, other entrepreneurs, etc.

Concrete suggestions were made, such as the demand to be supported by expert mentors who know how to steer the whole process according to young entrepreneurs' needs and bring them the necessary knowledge about trends and the market. Additionally, it was observed that they should be provided with knowledge regarding the disposal of adequate physical spaces that are cheap and well-located, roadmaps of supports and unique formats, and focused networking.

All this was coupled with the absence of social recognition for the entrepreneur, who often was not recognized but rather stigmatized. This argument reflects a cultural issue of the environment and is one of Isenberg's (2010) nine principles for building a better entrepreneurial ecosystem.

3) Strengths of the ecosystem

A common understanding of the existence of an entrepreneurial ecosystem was already considered a strength by itself, as in other regions, there is no such perception.

Other strong points identified were the capacity to support entrepreneurship by various means, both economically with money and humanly through people with ideas, institutional agents, and mentors—and there is also a business network since the business population in the territory is quite well-associated.

An interest in promoting entrepreneurial attitudes is not only reflected in the educational field—being more entrenched in the field of vocational training than in the university—but there were also programs, contests, and actions, etc. that encourage it as well. The presence of entrepreneurship in education comes from afar, which favors knowledge, prepared people, and referents of success, etc. that might constitute a guide to build on in the future.

4) Weaknesses of the ecosystem

For this topic, the atomization problem appeared again, as the existing shortcomings in terms of cohesion between support agents for entrepreneurship were mentioned, which means that there were duplications among them, as well as a lack of knowledge and information of the potential entrepreneur, who is the “client,” and about who is who within the ecosystem.

The table that had this topic assigned also discussed cultural matters, since they considered the business culture to be rather difficult, encompassing failures that are hardly forgiven and successes that are often criticized, which does not stimulate entrepreneurial activity. It does not encourage people, as to whether they did business wrong or right, they may be criticized and judged severely.

Finally, the ecosystem's entrepreneurial strategy was perceived as a weakness in three respects: entrepreneurship is understood as a tool to reduce unemployment, being preferably oriented toward youth entrepreneurship (knowing that the highest percentage of success in the venture is in age ranges of more than 35 years); and by wasting the potential of certain groups such as the self-employed or micro-SMEs, as those segments are capable of boosting successful entrepreneurship.

5) Ecosystem's challenges

The table working on this topic discussed the need to improve the attractiveness of entrepreneurship as a professional option. They considered that, at present, education through both university and professional training was more oriented to creating technicians, executives, as opposed to entrepreneurs. In this sense, the figure of the entrepreneur needs to achieve social recognition, which the participants consider as currently non-existent.

They also considered it interesting to encourage collaboration between potential entrepreneurs of different profiles, in favor of the development of an entrepreneurial project that, in its beginning, requires a capable team apart from financing the initiative. This was also related to Isenberg's (2010) suggestions.

The financing of venture initiatives was further commented on and was considered to be misfocused, as some initiatives aim to attract investors from outside who are not going to invest in local projects. It was, therefore, suggested to further enhance local investment. It was taken into account that a local investor, with the capacity to invest between 50,000 and 100,000 €, usually lacks the training necessary to understand certain new concepts that are linked to the ventures of startups and similar initiatives. This lack could be solved with training in how to invest in startups. It was suggested that investments should be changed in such a way that the venture capital funds avoid risks, especially in the case of seed capital, which is when entrepreneurs require their capital to access the funding that exempts them from risk. This prevents many projects from being able to access funds because they do not meet these requirements.

On the other hand, they also discussed the need to facilitate bureaucratic procedures for entrepreneurs and to unify the models of forms on the projects and business plans that must be presented, since the disparity and diversity of models that have to be adjusted cause them to not attend to their project for too long throughout the year. Hence, the problem of atomization and lack of coordination appeared again, another of the Isenberg (2010) principles for improving the ecosystem.

6 Role of collaborating agents, facilitators, or prescribers in the ecosystem

It was commented that there was a need for transparency among the agents that support entrepreneurship. Participants outlined that creating trust among agents was imperative and that it might result in a better performance in providing services to the final customer, the entrepreneur. In this sense, the visualization of the network was considered essential, and it had to be as extensive as possible in terms of the entities, services, and resources available. Thus, participants suggested using a platform where all this information is available as an objective or tangible part of the process, while the most important subjective or intangible part, should aim to enhance personal relationships, trust, and professionalism among agents.

All this should be incorporated without forgetting that once these networks have been created. They must be maintained and continue working, meaning that collaboration between agents through meetings or in ways that strengthen ties and allow greater coordination and efficiency of the work, is vital. The agents, who make up an entrepreneurial support system, should always bear in mind that their final objective should be to carry out the project of the entrepreneur.

### Co-creating Stage

The second session was about building together, generating ideas, and polishing those ideas during a co-creation process. In this case, the participants worked on six tables. All the participants were rotating between tables except for the one who was designated as the host and the facilitators who were part of the work team.

The work was divided into two sections. The first part of the dynamic was about detecting critical points or breaks during the seven stages, in which the life cycle of an entrepreneurial project was divided into: 1) awareness or entrepreneurial culture, 2) idea, 3) prototyping or business plan, 4) viability or financing, 5) constitution, 6) startup, and 7) consolidation.

Among the brakes detected was the stigma of failure, which was a restraint to entrepreneurial thinking associated with cultural bias. This stigma was perceived to undermine the possibility of assuming any type of risk from the start. In other cases, it stopped projects at the time of considering the startup. This perception of failure was considered to be deeply rooted in the collective consciousness, and came from school days, meaning it was a cultural factor. Not had this stigma been fought since school, it was also considered that there should be changes in the early stages of education, to instill risk-taking and the initiative to undertake projects to generate a critical mass of potential entrepreneurs who are now missing. However, addressing this problem involves two difficulties; on the one hand, teaching staff lack the knowledge and resources to impart the required qualities. On the other hand, family can also act as a hindrance to developing entrepreneurship because relatives might not consider entrepreneurship as a professional or desirable career for children. Concerning gaps in school education, it was suggested that collaboration with external experts in the field might alleviate this problem.

Another critical point was that as the projects progressed, there were shortcomings in the management knowledge of teams. Although technically the entrepreneurs were capable in their fields, they lacked other types of managerial skills.

Participants also outlined that they faced difficulties in achieving viable projects. They observed that it was difficult to materialize some ideas. Moreover, multidisciplinary and complementary teams were not always generated. Furthermore, they discussed how peers with shared experiences were missing, both in terms of successes and, perhaps more importantly, failures. In this sense, it was considered that small experiences or ventures were closer to inspirational means than those of large companies.

Participants also commented that they lacked a community of entrepreneurs, which could be understood as a lack of consciousness or a sense of ecosystem. Events that facilitate interaction between entrepreneurs could address this issue, allowing them to not only share common ideas and initiatives, meaning these events could be used to match different profiles. In this context, they would be able to test ideas and form teams. Participants were interested in physical spaces that fostered a favorable climate and encourages continuous entrepreneurial initiative.

Another point that arose was the resistance to sharing common ideas for fear of others stealing them. This means that the entrepreneurial team tends to validate the product before introducing it to the market, thus carrying the loss of many resources invested and making it impossible to pivot the project to try to test engagement in the market for the first time. This is a cultural matter.

Once the company was set up, there were financial shortages that hindered growth. Although, in some cases, this growth was hindered by the inability of the initial entrepreneurial team with projects ended because the team did not know how to manage success. There were also barriers to internationalization by the competencies of the team itself such as weak finance and management skills.

With regard to the support services offered by the network, participants mentioned that it was necessary to organize services, to make them known, and to encourage collaboration and synergies between different agents. Therefore, the problems arising from the atomization of the system were identified again.

Regarding aid, there were varying opinions. Some people perceived aid as necessary and others outlined that it was indispensable. The majority of opinions outlined that excessive focus on obtaining aid should be prevented and that the viability of the business should be perceptible without it. This could be related to the need to strengthen the roots of entrepreneurial initiatives, as suggested by Isenberg (2010). In the second part of the session, each table focused on developing two proposals to overcome the brakes that had been identified.

Regarding entrepreneurial culture, the participants suggested workshops (aimed at both parents and children) to change perceptions of failure, as an inevitable part of the process. These workshops could train and improve the skills of both students and teachers; and also provide spaces to create, with examples and close references that serve as inspiration and guides. Additionally, two suggestions were proposed: the launch of more idea contests and funding for the initial development of incipient ideas and projects.

Regarding the interaction among people, participants suggested the creation of physical spaces and events, which would allow the mix of all three key elements—minds, management, and money. The importance of the early validation of the project as well as the importance of the business model was also stressed.

In terms of support services related to the atomization problem, participants suggested that existing services and networking should be better coordinated and that there should be unified access or sources of information. It would be advisable for a mentor to accompany the team throughout the process with a memory briefing of what each agent contributed to that project, where the performance of these agents is measured by indicators for the services provided. In addition, business plans were suggested to not only ensure that the time of entrepreneurs accessing the system was not wasted, but also that the system should be oriented to the entrepreneur (which is supposed to be ecosystem and not “egosystem”).

### Landing Stage

To prepare the third session, which aimed to propose an action plan, the proposals of the preceding phases were narrowed and grouped into 14 areas that were randomly assigned to groups. Thus, the contributions developed in the session can be condensed into categories relating to the problems being addressed:

The first category regards the atomization problem:

1) Create a centralized gateway by concentrating online information and advice, simplifying processing, and facilitating a map of agents and aids

The use of social networks was proposed as a communicative vehicle linked to the creation or updating of a page or portal that agglutinates to the whole network, in which a map is displayed with all the available ordered information of the agents.

2) Coordination and collaboration between the agents working in a network, unifying forms, and creating new indicators for the measurement of performance.

It was proposed that the action be divided into three parts. First, the aforementioned map should be used, and segmented according to the different phases. This should be made known among the agents, allowing them to share information through the page, which would act as a collaborative platform *via* the web. For this, it is essential to have the commitment and the involvement of all. Secondly, it was considered that processes, objectives, and ways of measuring the results obtained should be coordinated. In this part, the decision of the government is fundamental since they are responsible for setting the measurements. Finally, aid needs to be optimized.

The second category is oriented to changes in mindset and the culture of the ecosystem to create a favorable environment that incentivizes new ventures and innovation:

3) Think globally by importing good practices and by contacting and collaborating with international networks aligned with state and community initiatives

For this, it would be interesting to share existing international contacts as well as organize expeditions or conferences for the dissemination of best practices. In addition, it would be necessary to have expert mentors from international markets. We should also share experiences and try to attract talent and international investments as well as create local consortia to act globally and make companies think at that level. It would be important to have an orderly resource map to avoid overlap and duplication.

4) Promote the entrepreneurial community by creating events and forums.

Numerous regular meetings, events, or forums for entrepreneurs would act as physical spaces or places in which they could share experiences or ideas.

5) To promote the generation of ideas by offering aid for the development of previous ideas and spreading new business models.

There were a wide variety of suggestions from generating thematic events, contests of ideas (including the school stage) involving professors, entrepreneurs, and companies, and sharing experiences through videos, lectures, and interviews, using both traditional media, social networks, and the Internet.

6) Change the perception of failure.

It was felt that it was important to perceive so-called failures as an inevitable part of any creative process. This should be taken into account when designing the support process *via* the use of mentors and also training. One approach might be to disseminate references for people who were wrong initially and how their failures led them to learn. It is also important that potential investors and public agents change their perceptions of failure.

7) Training: Differentiated training during the process and preparation of teachers.

It would be helpful to educate both entrepreneurs and the families of young people, raising awareness of the influence and the importance of these values and personal skills.

The third category is about strengthening projects, which includes both the configuration and the financing of the initiatives.

8) Accompaniment: mentoring or tutoring differentiated projects depending on the stage of the project.

It is important to accompany entrepreneurs at the beginning of the project and when they face failures. In addition to agents, it was considered important to generate meeting spaces between potential entrepreneurs and people who have gone through these experiences. To achieve this, it was considered necessary to have spaces or meeting forums.

9) Strengthening initiatives by promoting multi-disciplinarity and complementarity, and promoting teams to generate co-creative spaces that favor networking.

It was suggested that face-to-face and virtual encounters involving all kinds of agents would be helpful, and that big events that introduce activities for the promotion of entrepreneurial initiatives might also provide networking opportunities.

10) To insist on a rapid validation in the market by producing a prototype or developing a minimum viable product to be tested before the development process is complete.

It was proposed that creating a safe means to perform validation and involve experts in the field *via* the portal would be helpful.

11) Help to pivot if there is no engagement in the market or acceleration if it works and there is traction.

It was suggested to focus more on taking projects to action rather than on the planning stage and entrepreneurs and agents should be trained in techniques concerning product-market fit.

12) Facilitate funding by training local investors and encouraging their participation in projects.

Participants distinguished between capital or initial aid and financing, which, in both cases, can be public and private. For the first, it was proposed that they educate and sensitize the different groups that have funds to contribute. There should also be consultancy for both parties. It was also proposed that there could be financial incentives encouraging entrepreneurs to look for synergies and collaborations between different sources.

13) Encourage collaboration between investors and various sources of funding.

This resulted in three possible actions: the first was to cover gaps in financing in the initial phases of projects that require seed capital; the second, was to use the agent map to raise awareness of all possible existing sources; and finally, to promote knowledge and mutual encounters between investors through events that may facilitate opportunities for collaboration.

The fourth category regards governance:

14) Valuing entrepreneurial initiative: Showing and promoting the development of personal capacities.

It was proposed, first, to involve families and to act *via* education from early stages, which could generate activities and dynamics from school, and encourage a positive perception of learning from failure.

Each table organized the ideas they thought were more important in shaping the entrepreneurial network and presented them to the other groups.

## Conclusion

After 3 months of work and reflection *via* the three dynamics sessions discussed in this paper, which acted as milestones in January, February, and March 2018, this research indicated a clear need to create a formal network of entrepreneurial support. Participant discussions indicated that it needs to be a well-managed and coordinated subsystem, strengthened by an ICT-based platform. This would address the atomization problems identified by participants in the dynamic research sessions, who were a representative selection of all the stakeholders of the entrepreneurial ecosystem in the Basque Country.

This methodology involved committed agents and contributed to the birth of this network, allowing the achievement of the initial objectives of the study, addressing the needs of not only young but all entrepreneurs, since representative entrepreneurs took part in the dynamic sessions. Agents of support outlined the need for improved coordination, collaboration, and operation through a network with a shared entity supported by an online platform. A network would not only improve the interconnection between the agents but also act as a portal that gathers and connects potential entrepreneurs, for whom the support agents could provide services with added value.

A formal digitally reinforced network was identified as a solution to problems of atomization. In this network agents, entrepreneurs, resources, and services could be displayed, with a physical presence *via* events related to entrepreneurship. The network should consider all areas of action, including the education system, business structures, finance/investors, and public institutions, etc. The network would aim to foster a culture of entrepreneurship by naturalizing the failure inherent to this phenomenon, which should be understood as a process, and socially disseminate shared entrepreneurial experiences.

As a final output, and in response to the research discussed in this paper, the network was formally created in June 2018. And the use of an existing digital platform based on information and communication technologies (ICTs) was suggested and accorded; as it would allow public access and the location of all agents and their functions.

The next stage involved the launch of the network. That phase of development with measures and indicators is described by Cearra and Saiz (2021). This study concluded that the use of the ICT-based platform to boost the network was a key methodology. Its use enabled the researchers to measure the impact and results of the action quantitatively. Thus, the platform is a social network that improves the entrepreneurial ecosystem and also acts as a tool to evaluate public policies. We think that it could eventually be used as a proper tool to connect different entrepreneurial ecosystems.

This work has relevance to entrepreneurial ecosystem literature for several reasons. First, it is an empirical study addressing the so-called atomization problem and has the potential to enhance not only the coordination and collaboration among the support network but also to reinforce the relationship with the entrepreneurial people in the ecosystem. Second, it carries out a longitudinal study that will allow us to better understand the dynamics and consequent evolution as well as the impact the entrepreneurial ecosystem can have. Third, thanks to the use of the ICT-based platform, the measurement of the interactions will be easier than in previous research done in other locations.

Finally, this project led to the generation of social value, which was identifiable in several ways. First, by the proper co-creation process, which is social in nature and allows the participation of a large number of stakeholders present in the system. Second, because networks represent new governance models which are needed to align and improve the functioning and objectives of public and private stakeholders. Third, because this initiative could improve the entrepreneurial ecosystem, hence favoring entrepreneurial activity, which is conducive to new companies, employment, and essential in encouraging innovation to flourish. Fourth and finally, because innovation is crucial for accomplishing SDGs in the long term.

## Limitations and Future Research

This pilot project was undertaken in Biscay (Spain) and a clear limitation of the investigation is that it was based on a single region, meaning the results cannot be generalized. As a complex problem, a multiple case study methodology, as proposed by Yin (2014) might be suitable for future experiences.

A rigorous evaluation of the methodology demands the execution of the proposal outlined in the action plan at the start of this article. However, execution is not enough and as it is desirable to maintain the involvement and commitment of participants as time goes on, so responsibilities have to be fixed and development and must be controlled. Thus, a set of indicators need to be developed to act as a balanced scorecard, measuring the effectiveness of the project over time. Additionally, as with any experiment, new issues might arise during the extended research process, meaning this subject requires a longitudinal approach. Over time, these new issues also enhance understanding of the evolution of the entrepreneurial ecosystem, as Alvedalen and Boschma (2017) claim in their review about this research field.

Apart from measuring development at the network level, it is important to analyze the impact of this enhanced support network on the existing entrepreneurial ecosystem. Therefore, it is necessary to design a set of variables to evaluate the development of this project, standardize it, and share experiences with other ecosystems with similar atomization problems. There is still a lack of agreement about the set of indicators used to measure evolutionary dynamics in the literature about entrepreneurial ecosystems.

The methodology outlined in this study might be implemented in other geographical areas to test the application of these methods and whether they invigorate different entrepreneurial ecosystems. This challenge, as may other ones we have to confront, will require the involvement and participation of a wide variety of stakeholders to not only examine different points of view and perspectives on seeking feasible solutions but also to create a collaborative climate, so a similar approach might be used to address those issues.

## Data Availability Statement

The datasets presented in this study can be found in online repositories. The names of the repository/repositories and accession number(s) can be found at: https://www.dema.eus/wp-content/uploads/2019/12/red-sarekin-dema-bizkaia-castellano.pdf.

## Ethics Statement

The studies involving human participants were reviewed and approved by Bilbao Chamber of Commerce Research Committee. The patients/participants provided their written informed consent to participate in this study.

## Author Contributions

All authors listed have made a substantial, direct and intellectual contribution to the work, and approved it for publication.

## Funding

The Employment Department of the Provincial Council of Biscay financed and supported the design and application of this methodology and the development of the digital network (Sarekin) to implement it.

## Conflict of Interest

The authors declare that the research was conducted in the absence of any commercial or financial relationships that could be construed as a potential conflict of interest.

## Publisher's Note

All claims expressed in this article are solely those of the authors and do not necessarily represent those of their affiliated organizations, or those of the publisher, the editors and the reviewers. Any product that may be evaluated in this article, or claim that may be made by its manufacturer, is not guaranteed or endorsed by the publisher.
